# Multiple papular lesions in a patient with HIV and/or AIDS and coinfected with hepatitis B virus: Amyloidosis

**DOI:** 10.4102/sajhivmed.v18i1.735

**Published:** 2017-08-30

**Authors:** Hayati Demiraslan, Kemal Deniz, Mehmet Doganay

**Affiliations:** 1 Department of Infectious Diseases and Clinical Microbiology, Erciyes University, Turkey; 2 Department of Pathology, Erciyes University, Turkey

## Abstract

**Introduction:**

The most common form of systemic amyloidosis is amyloid A induced by a chronic inflammation. In HIV-infected patients, elevated serum amyloid A levels might be associated with chronic inflammation.

**Patient presentation:**

A 43-year-old male patient was admitted to hospital with a complaint of papular lesions around his eyes, existing for four months. The patient is receiving antiretroviral therapy. HIV RNA was undetectable, and the CD4 count was 770 cells/mm^3^. He suffered from a bladder carcinoma for four years. On examination, periocular, perioral and anogenital papules, papular lesions in the meatus of external auditory canal, and intranasal polyps were observed.

**Management:**

Microscopic examination of the biopsy material taken from the periocular lesion and then from perianal polyps revealed eosinophilic deposition, and stained positively by Congo red. Serum amyloid A level was negative. Antiretroviral therapy was continued.

**Conclusion:**

A rare form of amyloidosis in a patient with HIV and/or AIDS and coinfected with hepatitis B virus (HBV) was presented here with cutaneous and mucosal lesions.

## Introduction

The most common form of systemic amyloidosis is amyloid A induced by a chronic inflammation or chronic infections such as tuberculosis, chronic osteomyelitis, familial Mediterranean fever and rheumatoid arthritis. In HIV-infected patients, elevated serum amyloid A levels have been reported to be associated with chronic inflammation.^1,2^ HIV infection may cause amyloidosis by either direct action of HIV or its immunosuppressive effect.^3,4^ Clinical presentation depends on systemic organ involvement by amyloid fibrils, and the most common clinical manifestations proteinuria that leads to renal insufficiency.^5^ Here, a rare clinical presentation of amyloidosis in a patient infected with HIV was reported. Written informed consent was also taken from the patient, and the case report was approved by Erciyes University Local Ethics Committee (no: 352/2017).

## Patient presentation

**FIGURE 1:**
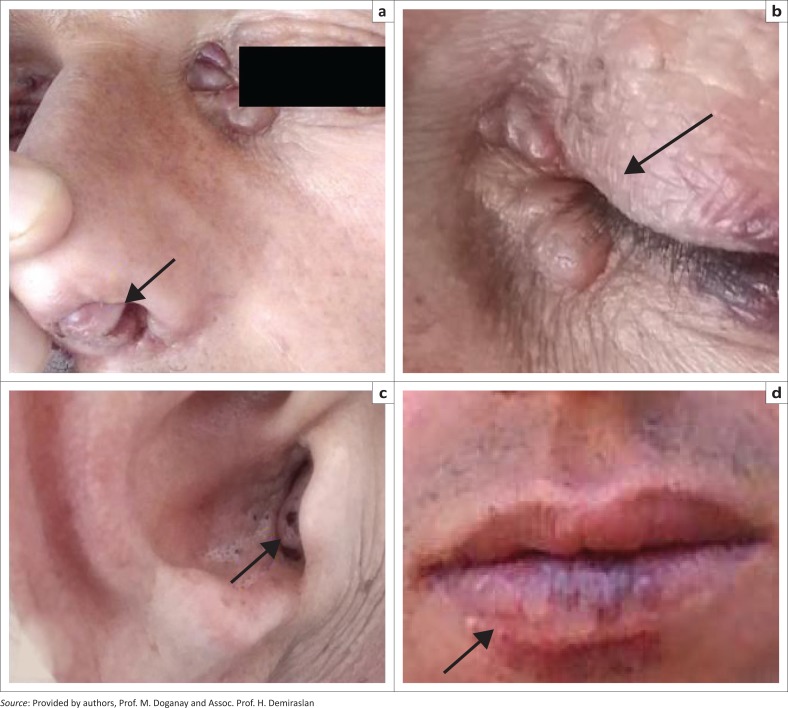
Appearence of (a, b) multiple nodular lesions around the eyes and within both nostrils (c) on the right external ear canal (d) and the lesions on the lower lip.

A 43-year-old male patient with HIV and/or AIDS was admitted to the Infectious Diseases Department for the investigation of papular lesions around his eyes in November 2014. The patient was diagnosed with HIV and/or AIDS and hepatitis B virus (HBV) coinfection in 2004 and antiretroviral therapy was initiated with a combination of lamivudine, zidovudine and lopinavir and ritonavir. This regimen was given between 2007 and 2010. Later, the therapy regimen was switched to tenofovir and emtricitabine and efavirenz. A bladder carcinoma was diagnosed in 2011 and he received mitomycin C in cycles. He denied fever and pain but sometimes complained of bleeding from his perianal polyp, and he claimed the lesion colour changed from pale to violet. On physical examination, periocular, perioral and anogenital papules and papular lesions in the external auditory canal and intranasal polyps were present. The lesions were lilac-coloured, soft and painless (Figure 1a, 1b, 1c and 1d). His body mass index was 21 kg/m^2^.

### Management and outcome

**FIGURE 2:**
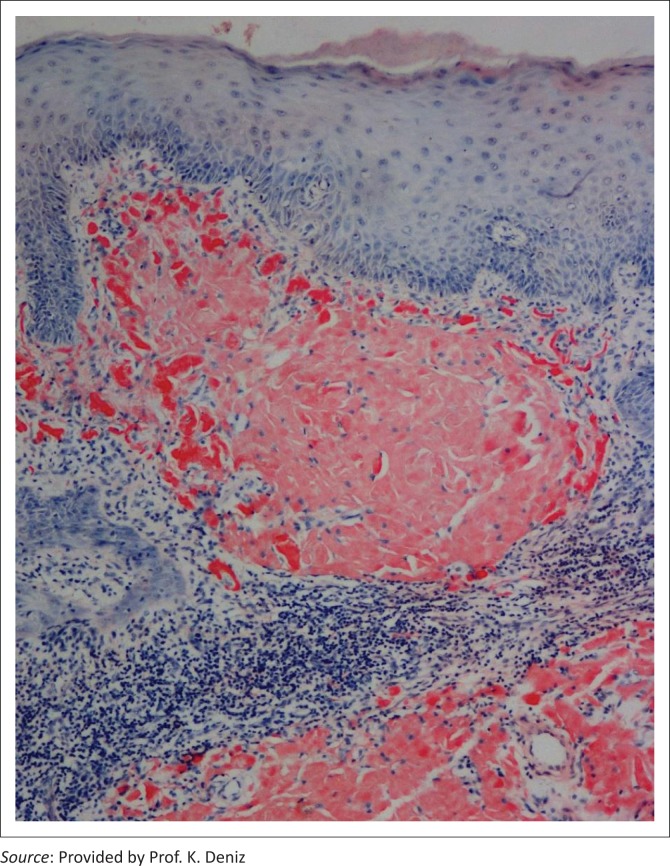
Photomicrograph showing typical amorphous extracellular Congo red–positive amyloid deposition within the dermis (Congo red, x100).

Routine blood count and biochemistry tests were within normal limits. Urinalysis showed microscopic haematuria and proteinuria (3 g/day). The creatinine clearance was calculated to be 80 mL/min according to the Cockroft–Gault equation. Abdominal ultrasound examination showed an echogenic stone of 8 mm diameter in the lower pole of the left kidney. The size of both kidneys was found to be within normal limits. A biopsy was performed from the periocular lesion in 2014 and from the perianal polyp in 2016. Histopathologic examination revealed eosinophilic deposition in the tissues and a positive staining with Congo red showing amorphous eosinophilic deposition (Figure 2). Apple green birefringence was visualised under polarised light. Serum amyloid A level was negative. Renal biopsy could not be performed because the patient refused the procedure. HIV RNA was undetectable (< 20 copies/mL), and the CD4 count was 770 cell/mm^3^. The patient is currently receiving an antiretroviral regimen consisting of dolutegavir and lamivudine, and additional oral colchicine therapy.

#### Ethical considerations

Verbal and written consent were taken from the patient for his clinical presentation and outcome. The case report was also approved by Erciyes University Local Ethics Committee (no: 352/2017).

## Discussion

Serum amyloid A is an acute-phase protein produced by the liver and it is related to high-density lipoprotein particles.^1^ One cause of systemic inflammation may be mitochondrial dysfunction, which is induced by nucleoside reverse transcriptase inhibitors.^6^ Amyloid may be induced by chronic inflammation and cancer.^1^ The presented case had HIV infection and HBV coinfection since 2004 and bladder carcinoma since 2011. Either of these might lead to secondary amyloidosis. A limited number of cases have been reported of secondary amyloidosis in HIV patients who had nephrotic syndrome.^7,8^ In this case, renal biopsy could not be performed because the patient refused the biopsy. The most common clinical presentation of amyloidosis is related to renal disease, which includes nephritic syndrome.^3,7,8^ Although this patient had nephrotic renal disease with proteinuria of 3 g/day; however, the patient’s main presenting complaint was related to cutaneous involvement periocular, intranasal and perioral papules.

## Conclusion

In conclusion, unusual presentations of amyloidosis with multiple cutaneous involvement may be seen in HIV patients especially complicated with cancer and HBV coinfection.
